# How new nanotechnologies are changing the opioid analysis scenery? A comparison with classical analytical methods

**DOI:** 10.1093/fsr/owae001

**Published:** 2024-01-05

**Authors:** Muhammad Usman, Yawar Baig, Donatella Nardiello, Maurizio Quinto

**Affiliations:** Narcotic Unit, Punjab Forensic Science Agency, Home Department, Government of The Punjab, Lahore-54000, Pakistan; Department of Sciences of Agriculture, Food, Natural Resources and Engineering (DAFNE), University of Foggia, I-71122 Foggia, Italy; Narcotic Unit, Punjab Forensic Science Agency, Home Department, Government of The Punjab, Lahore-54000, Pakistan; Department of Sciences of Agriculture, Food, Natural Resources and Engineering (DAFNE), University of Foggia, I-71122 Foggia, Italy; Department of Sciences of Agriculture, Food, Natural Resources and Engineering (DAFNE), University of Foggia, I-71122 Foggia, Italy

**Keywords:** forensic sciences, forensic toxicology, nanomaterials, chemical sensors, nanosensors, opioids, narcotic drugs

## Abstract

Opioids such as heroin, fentanyl, raw opium, and morphine have become a serious threat to the world population in the recent past, due to their increasing use and abuse. The detection of these drugs in biological samples is usually carried out by spectroscopic and/or chromatographic techniques, but the need for quick, sensitive, selective, and low-cost new analytical tools has pushed the development of new methods based on selective nanosensors, able to meet these requirements. Modern sensors, which utilize “next-generation” technologies like nanotechnology, have revolutionized drug detection methods, due to easiness of use, their low cost, and their high sensitivity and reliability, allowing the detection of opioids at trace levels in raw, pharmaceutical, and biological samples (e.g*.* blood, urine, saliva, and other biological fluids). The peculiar characteristics of these sensors not only have allowed on-site analyses (in the field, at the crime scene, etc.) but also they are nowadays replacing the gold standard analytical methods in the laboratory, even if a proper method validation is still required. This paper reviews advances in the field of nanotechnology and nanosensors for the detection of commonly abused opioids both prescribed (i.e. codeine and morphine) and illegal narcotics (i.e. heroin and fentanyl analogues).

## Introduction

**Figure 1:**
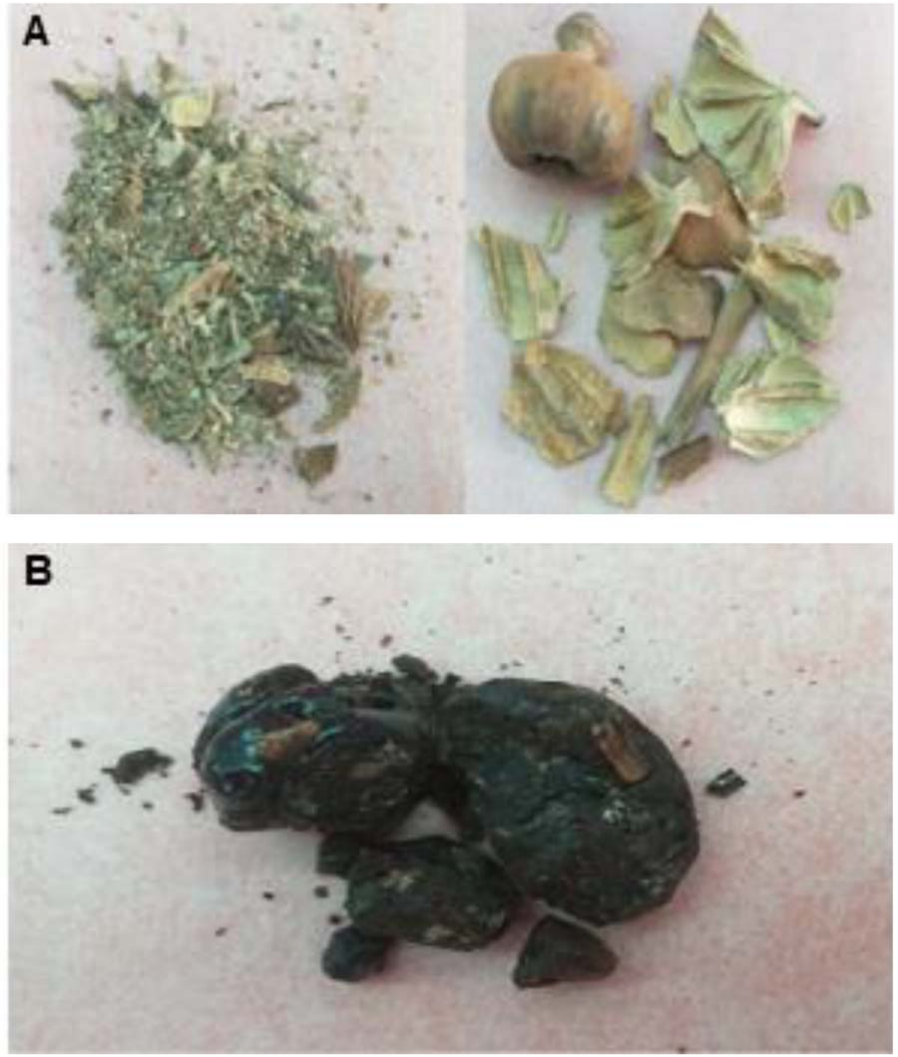
(A) Poppy plant material and (B) raw opium.

*Papaver somniferum* L. produces milky juice which is converted to dark brown resinous material after drying and is called raw opium (Figure 1), consisting of more than 30 alkaloids, resins, fatty acids, sterols, polysaccharides, triterpenoid alcohols, porphyroxine, meconic acid, and plant debris. Narcotic alkaloids of opium poppy are synthesized in specialized vesicles called “laticifers” which are found mainly in the stem. When some parts of the plant, especially the unripe pod, are incised, the milky latex oozes out, which is transported to the point of injury by laticifers and phloem tissues [1]. Out of 30 alkaloids, five are the main compounds and are classified into two categories, namely isoquinoline alkaloids (noscapine and papaverine) and phenanthrene alkaloids (codeine, morphine, and thebaine). Opium is not only used for illicit purposes (e.g. the synthesis of heroin from morphine) but also has legitimate applications. It is used for medicinal purposes, and for cooking, e.g. the seeds are used in bakery products (oil extracted from seeds) [2, 3] and in past, the oil obtained from opium was used in varnishes and paints [4, 5].

Opium is classified as a narcotic analgesic and is the only source of natural opioids. Since their origin, opioids are classified into three major categories, i.e. natural, semisynthetic, and synthetic. Codeine and morphine are natural opioids obtained from opium resin. Semisynthetic opioids are synthesized from natural opioids, like diacetylmorphine (heroin) which is the acetylated product of morphine [6]. Hydrocodone and oxycodone are also semisynthetic opioids. Fentanyls (whose pain-relieving capacity is 100 times greater than that of heroin) are synthesized in laboratories and classified as synthetic opioids. The lethal dose of fentanyl in humans is 2 mg. Non-pharmaceutical fentanyls (NPFs) are synthesized in clandestine laboratories and supplied to the drug market. Sufentanil, alfentanil, remifentanil, and carfentanil are the most common fentanyl analogues. Opioids have been used as pain relievers, antitussives, and sedatives. Opioids (e.g. codeine, morphine, oxycodone, heroin, dextromethorphan, pentazocine, dextrorphan, and norpethidine) affect the μ- and *k*-opioid receptors and decrease pain sensation by inhibiting the release of neurotransmitters [7]. A list of the most common opioids, their use, and legal status is reported in Table 1. Due to their psychotropic effects, opioids are widely abused, hence causing serious health issues. The oral ingestion of raw opium in high dose cause myocardial suppression [8]. About 209 million people around the globe used opioids in 2022 [9]. Since several people abuse these illicit drugs, the identification and quantitative determination of drugs are becoming more important. Nowadays, several advanced analytical techniques, such as gas and high-performance liquid chromatography (GC and HPLC, respectively), very often hyphenated with mass spectrometer (MS) detector, nuclear magnetic resonance (NMR) spectroscopy, capillary electrophoresis (CE), Raman spectroscopy, and Fourier transform infrared spectroscopy (FTIR), are being used for the analytical determination of opioids [10–16]. For forensic opioid analysis, different samples, i.e. biological (blood, urine, hair, nail, and exhumed body), pharmaceutical (tablets, injections), and street drug samples (street powder, clandestine tablets) are submitted and analyzed routinely [12, 17–29].

**Table 1:** List of the most common opioids and their use.

Opioids	Description	Pharmaceutical use	Legal status [49]
3-Monoacetylmorphine	Semisynthetic, inactive metabolite of heroin with weak affinity for μ-receptors	–	Prohibited, schedule I
6-Monoacetylmorphine	Semisynthetic, metabolite of heroin produced by acetylation of morphine	–	Prohibited
Acetylcodeine	Semisynthetic, by-product of acetylation of codeine in poppy resin	–	–
Acetylfentanyl	Synthetic, analogue of fentanyl and fifteen times more potent than morphine	Never been licenced for medical use	Illegal, not prescriptible, schedule I
Acrylfentanyl	Synthetic, analogue of fentanyl, and designer drug	Analgesic	Controlled, not prescriptible, schedule I
Alfentanil	Synthetic, analogue of fentanyl	Analgesic, used as anaesthesia	Controlled, schedule II
Buprenorphine	Synthetic and used to treat opioid addiction	Used to treat chronic pain	Prescription drug, schedule III
Carfentanil	Synthetic, analogue of fentanyl, 1 000 times more potent than heroin	Used in veterinary medicine	Controlled, schedule II
Codeine	Natural, constituent of poppy resin, less potent than morphine	Analgesic and antitussive	Controlled, schedule II
Dextromethorphan	Synthetic, approved for medical use in 1953	Cough suppressant	Pharmacy medicine
Dextrorphan	Synthetic	Antitussive	Unscheduled
Dihydrocodeine	Semisynthetic, derived from codeine, twice as strong as codeine	Analgesic and antitussive	Prescription drug, schedule II
Heroin	Semisynthetic, first derived from morphine in 1874	Analgesic	Prohibited, schedule I
Hydrocodone	Semisynthetic, derived from codeine	Analgesic, antitussive	Controlled, schedule I
Hydromorphone	Semisynthetic, derived from morphine	Used to treat severe pain	Controlled, schedule II
Methadone	Synthetic opioid	Used to treat chronic pain	Controlled, schedule II
Metopon	Methylated derivative of hydromorphone	Used to treat chronic pain	Controlled, schedule II
Morphine	Natural, constituent of poppy resin	Analgesic	Controlled, schedule II
Morphine-3-glucuronide	Metabolite of morphine	–	–
Morphine-6-glucuronide	Metabolite of morphine, stronger μ-receptors affinity than morphine	–	–
Nicocodeine	Semisynthetic	Analgesic and cough suppressant	Prohibited, not prescriptible, schedule I
Normorphine	Semisynthetic, derived from morphine; one-fourth as active as morphine	Analgesic	Prohibited, schedule I
Norpethidine	Synthetic, precursor in the synthesis of other drugs	Analgesic	Controlled, schedule II
Noscapine	Natural, constituent of poppy resin	Antitussive	Uncontrolled
Oxycodone	Semisynthetic, first derived from thebaine in 1916	Analgesic	Controlled, schedule II
Oxymorphol	Metabolite of oxymorphone; can be manufactured	Analgesic and cough suppressant	–
Oxymorphone	Semisynthetic	Analgesic, used to treat severe pain	Controlled, not prescriptible, schedule I
Papaverine	Natural, constituent of poppy resin	Antispasmodic	Prescription-only
Pentazocine	Synthetic, approved for medical use in 1964	Pain killer	Controlled, schedule IV
Pethidine	Synthetic opioid	Pain relieves	Controlled, schedule II
Pholcodine	Semisynthetic alkaloid	Opioid cough suppressant	Prescription drug in dose, schedule I
Remifentanil	Synthetic, analogue of fentanyl, used for general anaesthesia	Pain relievers and anaesthetic	Controlled, schedule II
Sufentanil	Synthetic, analogue of fentanyl, 500 times more potent than morphine	Analgesic, used as anaesthesia	Controlled, schedule II
Thebaine	Natural, constituent of poppy resin	–	Controlled, schedule II
Tilidine	Synthetic opioid	Pain killer	High-dose prescription drug, schedule I
Tramadol	Synthetic opioid	Pain relieves	Prescription drug, schedule IV

Nanomaterial-based techniques have been employed for the determination of opiates, offering innovative approaches with high sensitivity and versatility. In this review, new analytical methods based on nanomaterials used as biochemical sensors or electrochemical sensors for qualitative and quantitative analysis of opioids have been discussed and compared with classical analytical determinations. Chemical sensors based on nanomaterials have applications in the determination of opioids in a variety of samples [30–32]. Carbon nanotubes (CNTs), like single-walled CNTs (SWCNTs) or multi-walled CNTs (MWCNTs) [33, 34], graphenes [35], tungsten oxide nanoparticles [36], palladium nanoparticles [37], platinum nanoparticles [38], gold nanoparticles (AuNPs) [39], and other modified nanomaterials have been used as sensors for opioids and their extraction from biological samples [31, 40]. CNTs are well-known for their good biocompatibility and electrical sensitivity, and their applications in biosensing are numerous [41, 42]. CNTs have been widely used in sensor preparation for the analysis of electro-active drugs [43]. When deposited on metal electrodes, SWCNTs, MWCNTs, and CNTs can be used as chemical sensors. Graphene, composed of layers of tightly bound carbon atoms (honeycomb network), nanomaterial is also used as a chemical sensor or in the construction of modified electrodes [44]. Various types of electrodes, i.e. glassy carbon electrode [45], carbon paste electrode [46], indium tin oxide electrode [47], Pt electrode [48], and carbon ceramic electrode [34] are combined with amperometry, voltammetry, or impedance spectrometer for drug analysis. The chemical sensors have several advantages over the other techniques, i.e. low cost, portability, shorter time of analysis, high sensitivity, and low limits of detection. These advantages make chemical sensors a very attractive material for future research.

### Classical analytical methods for the detection of opioids

#### Colourimetric assays

Colour testing assays are based on the generation of colour by specific chemical/biochemical reactions between target analytes and reagents. Therefore, colour tests produce simple visual results (i.e. change in colour), and complex instrumentation is not required for the analysis. Colour tests are rapid detection tools for qualitative determinations and can be used to evaluate the presence or absence of drugs in a test sample, outside the controlled laboratory environment. Nevertheless, false positive and false negative results represent a major concern for analysts during drug analysis, since the presence of interfering compounds in biological samples may negatively affect the analytical results. For this reason, colour tests are generally used in combination with other techniques (e.g. GC–MS, LC–MS, or FTIR). A schematic overview of the main tests used for presumptive screening of opioids in suspected drug samples, namely, Marquis, Froehde, Meck, nitric acid, Oliver, Mandelin, Liberman, and AuNPs tests [50, 11, 25, 51, 52], is given in Table 2.

**Table 2:** Colour tests used for preliminary opioid screenings.

Test name	Analyte	Colour expression	Reference
Marquis	Codeine, morphine, heroin	Violet, reddish-purple	[53]
Froehde	Codeine, morphine, heroin	Purple, green	[54]
Meck’s test	Codeine, morphine, heroin	Blue, green	[55]
Nitric acid	Heroin	Red-orange, green	[56]
Oliver	Heroin	Pink, red	[57]
Mandelin	Codeine, heroin, morphine	Blue-green, blue-grey, light grey	[53]
Lieberman	Codeine, morphine	Black	[53]
AuNPs	Codeine	Green	[51]

#### Spectroscopic methods

Spectroscopic techniques are frequently applied in forensic investigations for the analytical determination of controlled substances. However, according to the protocols of the Scientific Working Group for the analysis of seized DRUGs (SWGDRUG), chromatographic techniques have to be employed in addition to spectroscopic techniques (i.e. UV, IR, FTIR, Raman Spectroscopy, and Mass Spectrometry) [16, 58, 59]. Spectroscopic instruments are generally user-friendly and require a very minimum amount of sample. Recently, portable spectroscopic instruments have been developed, which make these techniques ideal for field testing and on-spot analysis [60]. A portable ultraviolet Raman Spectroscopy device, developed by Hopkins et al. [61], was successfully applied for the determination of heroin [61, 62]. By terahertz spectroscopy, it is possible to obtain the structural and chemical information of substances concealed within packaging (e.g. cardboard, plastic, and paper). Due to this feature, terahertz spectroscopy can also be used for on-spot street drug identification. Terahertz time-domain spectroscopy (THz-TDS) shows characteristic peaks at 1.42 and 3.94 THz for heroin [63]. In addition to field testing of drugs, spectroscopic techniques have been applied for component analysis and drug profiling. FTIR-ATR has been used for the qualitative and quantitative determination of morphine and three commercial variants of thebaine in poppy extracts [64]. Surface-enhanced Raman spectroscopy [65, 66], diffuse reflectance near-infrared spectroscopy (DR-NIR) [67], and near-IR Raman spectroscopy [68, 69] have been also reported for the analytical determination of natural and synthetic opioids.

#### Chromatographic methods

Chromatography is the most used and widely accepted analytical technique amongst the forensic community. GC–MS [11, 12, 20, 70], or tandem mass spectrometry (GC–MS–MS) [18], and flame ionization detection (GC-FID) [71] have been reported for the determination of illicit drugs in human-origin biological samples. A quick, economical and eco-friendly method by dispersive liquid–liquid micro-extraction (DLLME) coupled with injection port silylation-gas chromatography–mass spectrometry (IPS-GCMS) was developed and validated by Jain et al. [72] for the determination of morphine in illicit opium, using bis(trimethylsilyl) acetamide for the derivatization process of morphine. For the analysis of non-volatile and higher molecular weight drugs, liquid chromatography coupled to mass spectrometry (LC–MS) [17, 23, 73] and ion mobility tandem mass spectrometry (LC–TIMS–MS) [74] has been employed. Recently, UHPLC–MS–MS has been reported for the determination of synthetic opioids including fentanyl in hair samples [75]. Lui et al*.* [76] proposed an ultra-performance liquid chromatography method coupled with quadrupole time-of-flight mass spectrometry (UPLC-Q-TOF) for the characterization of opium samples from different geographical sources. Mass spectrometry-based chromatographic methods allow accurate qualitative and quantitative multi-residual determinations of drugs at the trace level in a variety of samples, providing high analysis selectivity and sensitivity. Nevertheless, GC–MS and LC–MS methods are generally time-consuming and labour-intensive and require sophisticated equipment for exclusive laboratory-level use [77, 78]. As an alternative to mass spectrometry, GC coupled with vacuum ultraviolet spectrometry (VUV) has been used for the determination of opioids (i.e. morphine, heroin, and fentanyl). The specificity of VUV spectra was evaluated by using principal component analysis and discriminant analysis (DA) statistical techniques, then the GC-VUV method was successfully applied for the analysis of street heroin samples with 100% accuracy of the DA model [79]. A list of the most common methods used for the determination of opioids/opioids is reported in Table 3.

**Table 3:** List of drug abuse analyses carried out by classical techniques, with the indication of analyzed opioids/opioids, matrices, limit of detections (LODs), and limit of quantitations (LOQs).

References	Drug abuse analyzed	Technique	Sample	LOD	LOQ
[20]	Morphine 6-MAM Codeine	GC–MS	Liver, kidney, hair, and teeth	–	–
[70]	6-MAM	GC–MS	Bone	0.1 ng/mg	0.3 ng/mg
	Morphine			0.3 ng/mg	1 ng/mg
	Tramadol			0.2 ng/mg	0.5 ng/mg
	Methadone			0.3 ng/mg	0.8 ng/mg
[82]	Methadone	GC–MS	Hair	0.05 ng/mg	0.16 ng/mg
[83]	Methadone	GC–MS	Hair	0.15 ng/mg	0.36 ng/mg
[12]	Heroin	GC–MS	Street sample	–	–
[84]	Morphine Codeine 6-MAM Methadone	GC–MS	Hair	0.016 ng/mg 0.016 ng/mg 0.015 ng/mg 0.016 ng/mg	0.046 ng/mg 0.045 ng/mg 0.043 ng/mg 0.042 ng/mg
[85]	Fentanyl	GC–MS	Plasma	0.03 ng/mg	0.1 ng/mg
[86]	Morphine	GC–MS	Blood	2 ng/mg	10 ng/mg
	Codeine				
	6-MAM				
	Hydrocodone				
	Hydromorphone				
	Oxycodone				
	Oxymorphone				
[87]	Morphine	GC–MS	Urine	10 ng/mg	25 ng/mg
	Codeine				
	6-MAM				
	Hydrocodone				
	Hydromorphone				
	Oxycodone				
	Oxymorphone				
[18]	Codeine Morphine 6-MAM Hydrocodone Hydromorphone	GC–MS/MS	Blood	1 ng/mg for all 2.5 ng/mg for hydrocodone	2.5 ng/mg for all 5 ng/mg for hydrocodone
[79]	Morphine, 3-MAM 6-MAM Heroin Codeine	GC-VUV Vacuum ultraviolet spectrophotometry	Street drug samples	–	–
[88]	Heroin	GC-FID	Street drug samples	0.02 mg/mL	0.05 mg/mL
[89]	Morphine	LC–MS/MS	Blood	16 ng/mg	
[90]	Morphine Codeine Methadone	LC–MS/MS	Serum, urine and post-mortem blood	0.01–1.7 ng/mg	0.04–4.2 ng/mg
[91]	Fentanyl derivatives and metabolites	LC–MS/MS	Oral fluid	0.05–0.50 ng/mg	0.1–1.0 ng/mg
[92]	Morphine	LC–MS/MS	Hair	2.0 pg/mg	5.0 pg/mg
	Codeine				
	6-MAM				
[93]	Morphine Codeine 6-MAM	LC–MS/MS	Oral fluid	0.8–2 ng/mg	2–5 ng/mg
[94]	Morphine	LC–MS/MS	Hair	3.8 pg/mg	12 pg/mg
	Codeine			4.7 pg/mg	16 pg/mg
	6-MAM			4.2 pg/mg	14 pg/mg
[23]	6-MAM	LC–MS/MS	Hair	25 pg/mg	100 pg/mg
	Morphine			5.0 pg/mg	10 pg/mg
	Codeine			2.0 pg/mg	10 pg/mg
	Hydromorphone			1.0 pg/mg	5 pg/mg
	Hydrocodone			4.0 pg/mg	10 pg/mg
[95]	Buprenorphine	LC-DED	Plasma	0.08 ng/mg	
	Norbuprenorphine			0.08 ng/mg	
	Naloxone			0.04 ng/mg	
	Methadone			0.9 ng/mg	
[58]	Methadone	HPLC-MS/MS	Postmortem samples	2.5 ng/mg	5 ng/mg
[26]	Morphine	HPLC	Street samples	1 ng/mg	5 ng/mg
[96]	Morphine	HPLC-PDA	Postmortem urine	10–25 μg/L	-
	Codeine				
	Methadone				
[75]	Fentanyl Furanylfentanyl Oxycodone Hydrocodone Tramadol Morphine 6-MAM Codeine	UHPLC–MS/MS	Hair	0.1–0.3 pg/mg for all 1.5 pg/mg for oxycodone	0.3–0.9 pg/mg for all, 1.5 pg/mg for noroxycodone
[97]	Morphine Codeine 6-MAM	HPLC-DAD	Hair	–	0.2 ng/mg
[98]	Ocfentanil	UHPLC–MS/MS	Postmortem body samples	0.03 ng/mg	0.10 ng/mg
[99]	Heroin	ICP-MS	Street drug	–	–
[100]	Heroin	ICP-MS	Street drug	0.001–100 ng/g	0.1–500 ng/g
[101]	Heroin	ATR-FTIR	Street drug	–	–
[67]	Heroin	DR-NIR	Street drug	–	–
[102]	Heroin (characterization)	^1^H NMR	Street drug	–	–
[14]	Codeine Tramadol	Capillary Electrophoresis	Pharmaceutical preparations	15 μmol/L 0.62 μmol/L	–

MAM: monoacetylmorphine; GC: gas chromatography; MS: mass spectrometry; VUV: vacuum ultraviolet spectrometry; FID: flame ionization detection; LC: liquid chromatography; DED: dual electrode detection; HPLC: high-performance liquid chromatography; PDA: photodiode array; UHPLC: ultra high performance liquid chromatography; DAD: diode-array detection; ICP: inductively coupled plasma; ATR-FTIR: attenuated total reflectance Fourier transform infrared; DR-NIR: diffuse reflectance near-infrared spectroscopy; NMR: nuclear magnetic resonance.

### Sensors for the determination of opioids

#### Electrochemical sensors based on nanomaterials

Nanomaterial-modified electrochemical sensors have been used for the detection of opioids. The use of nanomaterials for the synthesis of electrochemical drug sensors attracted the interest of scientists because they can be employed for rapid and on-site qualitative determination of illicit drugs in different substrates [80]. There are numerous advantages to using electrochemical sensors due to their sensitivity, selectivity, specificity, and easiness of use, even with complex biological media, like human urine and pharmaceutical samples [41]. Electrochemical sensors are classified into three categories on the bases of their sensing mechanism: amperometric, conductometric, and potentiometric. Usually, electrode material is specifically developed for a target drug. Electrochemical sensors show a good linear range response and high selectivity, but they suffer from a slow response. Ensafi et al. [38] developed an electrochemical sensor based on Pt nanoparticles supported on porous silicon flour for the simultaneous determination of morphine and codeine, using adsorptive stripping voltammetry. Detection limits of 30.0 and 20.0 nmol/L were achieved for morphine and codeine, respectively. The electrochemical determination of morphine was achieved by properly modifying carbon paste electrodes [81]. Differential pulse voltammetry (DPV) was applied to a carbon paste electrode modified with multiwall carbon nanotubes (MWCNTs) for drug determinations. Cyclic voltammetry, chronoamperometry, and electrochemical impedance spectroscopy were used by Mokhtari et al. [103] with modified carbon paste electrode with vinylferrocene/MWCNTs to study the response as a function of morphine presence in biological and pharmaceutical samples. Gold nanoparticles, deposited on nafion, can be used for modification of carbon paste electrodes to detect morphine in urine samples using DPV [104]. A carbon ceramic electrode was modified by SWCNTs for the simultaneous detection of codeine and caffeine using DPV [34]. Atta et al. [105] applied a modified gold electrode with gold nanoparticles over poly(3,4-ethylene-dioxythiophene) for the determination of morphine in urine and pharmaceutical tablets, using sodium dodecyl sulphate (SDS) as a surfactant. A carbon glassy electrode modified by reduced MWCNTs-doped graphene oxide was developed for the analysis of morphine. The electrochemical response of morphine was investigated using pH 4.5 phosphate buffer solution by cyclic voltammetry and linear sweep voltammetry [106]. A similar electrochemical sensor was developed by Babaei et al. [43]: they modified a glassy carbon electrode by MWCNTs/chitosan for the detection of dopamine and morphine in human blood and urine. Cyclic voltammetry, DPV, and chronoamperometry were used for the analysis of drugs using the above-mentioned sensor, obtaining a sensitive amperometric sensor for morphine detection, which exhibited high sensitivity, stability, and long life. Heroin, morphine, and its metabolite morphine-3-glucuronide can be also detected by amperometric devices, properly synthesized for this scope [45, 47, 107–109]. Tey et al. [6] synthesized a disposable device for onsite drug screening based on a liquid-gated carbon nanotube field effect transistor (LG-CNTFET), for the detection of 6-acetylmorphine (a heroin metabolite). The detection limit of LG-CNTFET has been improved by incorporating gold nanoparticles into morphine antibodies. El-Naby and Kamel AH [110] developed a potentiometric assay method for the determination of dextromethorphan in pharmaceutical preparations and illicit drug market samples. The membrane-based polymer, composed of molecularly imprinted polymer (MIP) with methacrylic acid (MAA) and acrylonitrile (AN) acting as functional monomers, was developed, and the relevant biosensor characteristics have been fully validated, showing short response time, high sensitivity, selectivity, stability, and accuracy. The limit of detection (LOD) of MIP/MAA and MIP/AN sensors were 1.9 × 10^−6^ and 1.0 × 10^−6^ mol/L, respectively.

Metal–organic frameworks (MOFs) and their composites have emerged as pivotal players in the development of electrochemical sensors dedicated to opioid detection. Renowned for their rapid production, cost-effectiveness, high sensitivity, and remarkable low detection limits, these materials represent a paradigm shift in sensor improvement. Their intrinsic potential in facilitating real-time drug concentration measurement, a critical aspect for tailoring treatment dosages to uphold therapeutic thresholds, is particularly important in the clinical application of opioids. Furthermore, the integration of microfluidic chips with electrochemical sensors can enhance detection capabilities [111].

Razavi et al. [112] developed a CuONPs/MWCNTs/carbon paste modified electrode for tramadol analysis. Employing eco-friendly synthesis, cupric oxide nanoparticles (CuONPs) were crafted using *Origanum majorana* extract, revealing perfect structures, as verified through X-ray diffraction (XRD), SEM, and FTIR. Voltammetric methods highlighted the electrode’s precision, demonstrating exceptional selectivity for tramadol and establishing accurate linear calibration curves. Cutting-edge techniques based on B3LYP/LanL2DZ quantum method were used to calculate the energy characteristics of the nanocomposites. CuONPs/CNT demonstrated effectiveness in detecting Tramadol in actual samples with a recovery rate of 96.0%–104.3%.

A novel electrochemical platform, integrating a covalent organic framework (COF) and reduced graphene oxide (rGO), was successfully devised for the highly sensitive detection of fentanyl and alfentanil in human serum. The COF nanomaterials, exhibiting a distinctive chrysanthemum morphology, significantly increase electrochemical catalytic reactions, enhancing the overall sensor performance. Concurrently, the introduction of reduced graphene oxide increased the sensitivity by facilitating electron transfer. Under optimized conditions, the developed electrode presents a linear detection range of 0.02–7.26 μmol/L for alfentanil and 0.10–6.54 μmol/L for fentanyl, with impressive detection limits of 6.7 and 33.0 nmol/L, respectively. The sensor showed outstanding selectivity, reproducibility, and stability, positioning it as a viable tool for the reliable monitoring of fentanyl and alfentanil in human serum samples [113].

An innovative electrochemical sensor for the detection of tramadol, using a UiO-66-NH_2_ metal–organic framework (MOF)/third-generation poly(amidoamine) dendrimer (G3-PAMAM dendrimer) nanocomposite on a glassy carbon electrode, was developed. Various characterization techniques, including XRD, energy-dispersive X-ray spectroscopy, field emission-scanning electron microscopy (FE-SEM), and FTIR spectroscopy, confirmed the successful functionalization of UiO-66-NH_2_ MOF by G3-PAMAM. The UiO-66-NH_2_ MOF/PAMAM-modified electrode demonstrated commendable electrocatalytic performance for tramadol oxidation, exhibiting enhanced sensitivity within a broad concentration range (0.5–500.0 μmol/L), as revealed by DPV. These sensors maintained stability, repeatability, and reproducibility, even in the presence of acetaminophen, showing acceptable catalytic behaviour with a separated oxidation potential (*Δ*E = 410 mV). Furthermore, practical applicability was successfully demonstrated in pharmaceutical formulations, particularly in tramadol and acetaminophen tablets [114].

Fentanyl and its analogues (powerful synthetic opioids and the primary cause of drug overdose deaths in the United States) pose a critical challenge for their analytical determination. Canoura et al. [115] developed a series of innovative aptamer-based assays and electrochemical sensors, which offer reliable, accurate, rapid, and cost-effective means to detect fentanyl-based compounds. These sensors show no cross-reactivity with other illicit drugs or substances, even in interferent-laden mixtures containing only 1% fentanyl. The potential for routine use extends to medical professionals, law enforcement agencies, and the general public, facilitating swift and precise identification of fentanyl.

In recent developments within nanomaterials-based electrochemical sensors, significant progresses have been made in the analytical determination of tramadol, a centrally acting analgesic utilized for treating various pain conditions. The application of distinct electrochemical techniques, including DPV, amperometry, and SWV, has yielded noteworthy outcomes in the realm of tramadol detection by using MWCNTs-AuNPs, CoO-CNTs, and graphitic carbon nitride nanomaterials. Notably, these sensors exhibited a broad spectrum of detection limits and linear ranges, underscoring their versatility across applications. The integration of metal nanoparticles like AuNPs and metal oxide NPs, exemplified by Co_3_O_4_ NPs and CuONPs, has substantially enhanced the sensitivity of tramadol detection. Additionally, the utilization of diverse composites such as Nafion/CTAB-Au and Yb_2_O_3_-SPE underscores the variety in sensor fabrication approaches. Overall, these sensors demonstrated promising sensitivity over a wide concentration range, highlighting their potential for practical applications in detecting tramadol and emphasizing the pivotal role of nanomaterials in advancing electrochemical sensing technologies for pharmaceutical analyses [116].

#### Nano-biosensors

Nano-biosensors are an effective tool for the analysis of drugs, due to their selectivity which is usually greater than those of traditional biosensors. They convert biological response to electrical or optical output response through a bio-receptor that identifies the analyte and a transducer, which converts the signal to an electrical or optical output. Nano-biosensors for the analyses of illicit drugs are of great interest, not only in forensic investigations but also in monitoring drug abuse in treatment centres and workplaces, due to their unmatched properties (high selectivity, reproducibility, stability, sensitivity, affordability, linearity of response, low cost, and easy functioning). Nano-biosensor can be subdivided, depending on the bio-receptor and the transducer used, into immunosensors, genosensors, aptasensors, and electrochemical biosensors [117–121, 122, 123]. Genosensors obtained by recombinant DNA-based techniques have been used recently to produce antibodies for morphine-3-glucuronide (a metabolite of heroin). For example, an SPR-based inhibition immunoassay using anti-morphine-3-glucuronide recombinant scFv (single chain fragment variable) antibodies in *Escherichia coli* has been reported [124]. The response of antibody binding to the surface of the chip was inversely proportional to the amount of free drug in urine. Similarly, it was found that AuNPs labelled scFv antibodies could be used for the detection of morphine [125]. The developed recombinant antibodies bind morphine and monoacetylmorphine (MAM) very specifically. Double-stranded DNA (ds-DNA) immobilized on gold electrodes modified with mercapto-benzaldehyde was employed for sensing morphine [126]. The electrostatic interaction of morphine with ds-DNA was studied by DPV. Another biosensor was prepared on a pencil graphite electrode (PGE) modified with MWCNTs dispersed in poly(diallyldimethylammonium) (PDDA) [127]. In this case, the biosensor sensitivity is assured by conformational changes produced by codeine and morphine on ds-DNA, which provides variations on the electrochemical oxidation signals for both compounds. Differential pulse (DP) voltammograms of codeine and morphine showed depressed currents in the presence of DNA shifting to more negative and positive potentials, respectively. The electrostatic attraction for codeine and the intercalation for morphine permits clear discrimination between the peaks for both analytes. Voltammetric analysis, with an accumulation time of 5 min, provided a linear dependence between 0.05 and 40 mg/mL for codeine and 0.05 and 42 mg/mL for morphine, with detection limits of 0.041 and 0.043 mg/mL, respectively. This biosensor was successfully used for the analysis of blood, urine samples, and pharmaceutical preparations. Shao et al. [128] developed an exceptionally sensitive norfentanyl sensor, using a semiconductor-enriched single-walled carbon nanotube (sc-SWCNT) field-effect transistor modified with norfentanyl antibodies. Motivated by the pervasive global opioid crisis (particularly the impact of synthetic opioids like fentanyl), the study strives to meet the pressing demand for a portable analytical tool. Various sensor configurations have been explored, covering a direct coupling strategy and an approach incorporating gold nanoparticles (AuNP), with sensitivity optimization through the integration of a “reduced” antibody. The sensor exhibited notable sensitivity, achieving a 2.0 fg/mL LOD for norfentanyl in synthetic urine samples. Nanomaterials possess unique characteristics, such as high surface area, high conductivity, enhanced electron transfer rate, and ease of modification, which enable diverse applications, including sample pretreatment and biosensor establishment, facilitating the monitoring of illicit drugs in diverse complex matrices, including biological, pharmaceutical, and environmental samples [121, 129, 130]. Furthermore, the integration of nanomaterials in electrochemical sensors is advantageous, particularly for the detection of drugs present at low concentrations in body fluids. These sensors are also beneficial for on-site screening, as they enable non-invasive sample collection (allowing, as an example, drug detection in saliva samples), not requiring invasive blood sample collection [130]. Nanomaterial-based aptamer sensors have been used for the analytical determination of illicit drugs, showing higher sensitivity, and sensitivity than traditional methods. In addition, nanomaterial-based sensors require minimal sample pretreatment, have fast response times, and show potential for on-site analysis [131]. These sensors require minimal sample pretreatment, enabling fast and efficient analysis of complex matrices such as human urine, blood serum, and saliva [132]. They typically require less sample consumption (in some cases ~100 μL) contributing to potential cost savings in terms of sample usage; moreover, the utilization of nanomaterials provides superior detection efficiency, with limits of detection in the range of pg/mL and ng/mL [129]. Nanomaterial-based sensors are affordable, easy to use, and portable, making them suitable for a wide range of applications, from health monitoring to more complex settings, such as border customs or aviation industries [132]. On the other hand, these devices showed some limitations due to their drug specificity, i.e. it is necessary to use different sensors to detect different drugs [133]. The development of nanosensors can be complex, requiring expertise in nanotechnology and nanosensor design. This complexity may limit the widespread adoption of nanosensors for opiate analytical determinations, especially in settings with limited resources [121]. Whilst nanosensors offer high sensitivity and selectivity for the detection of opioids, ensuring specificity for different opiates can be challenging. Therefore, the development of nanosensors with high selectivity for various opiates remains an ongoing area of research [134].

#### Supramolecular sensors

**Figure 2:**
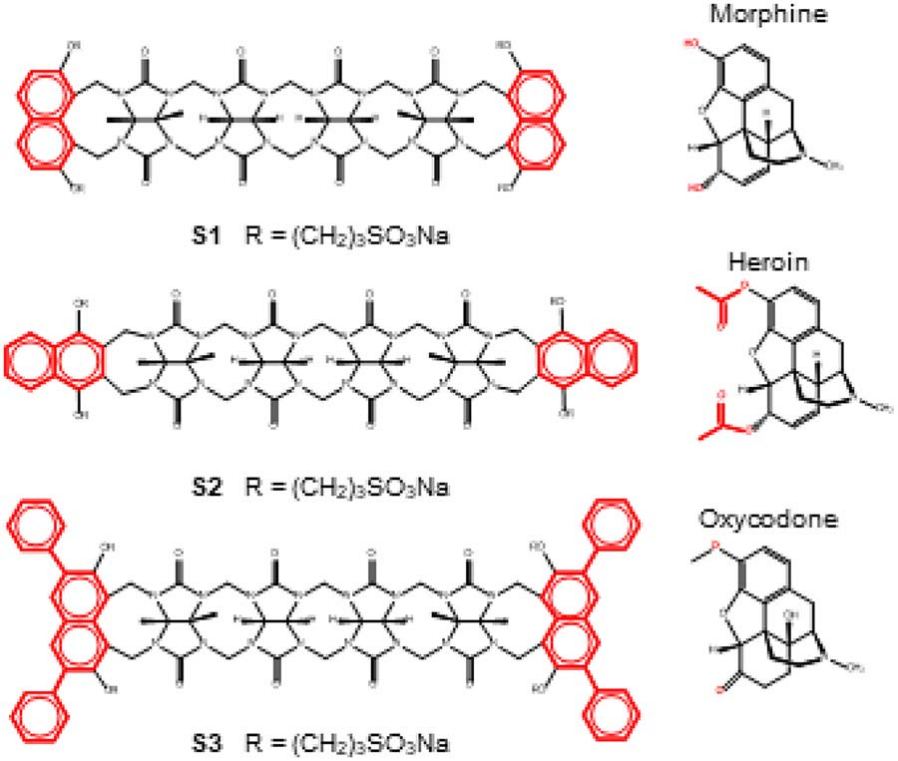
Chemical structures of supramolecular sensors (S1– S3) used for the analysis of opioids and their metabolites.

Supramolecular chemistry is a relatively young branch of science. In supramolecular sensors, the sensor molecule is selected based on the size, shape, and charge of the drug molecule. Sensor molecules contain binding sites, which are highly specific for drugs. The analyte molecule binds with the sensor at a specific binding site due to non-covalent forces, such as hydrogen bonding or Van der Waal forces. As a result of these attractive forces between the sensor and drug molecule, a supramolecular complex is produced. The stability of this complex depends on the strength of these non-covalent forces. The formation of supramolecular complexes can generate colourimetric, fluorescent, or electrochemical signals. Due to their high specificity, supramolecular sensors (Figure 2) are conveniently used in analytical chemistry for the determination of a variety of substances in complex samples. Acyclic cucurbituril (aCB) has been used and reported as a sensor molecule for the quantitative analysis of three opioids, i.e. morphine, heroin, and oxycodone, together with their metabolites normorphine, morphine-3-glucuronide, morphine-6-glucuronide, 6-monoacetylmorphine, noroxycodone, and oxymorphone [159]. aCB is composed of four glycoluril units that terminate on both ends with naphthalene fluorophore units. The cucurbituril opening cavity is hydrophobic and flexible, and its diameter depends on the connectivity between naphthalene rings and glycoluril units. Due to its flexibility, the cavity is adaptable to the shape of the opioid molecule. Intermolecular naphthalene acts as a chromophore, producing the desired fluorescence in the presence of the analyte. This sensor has been successfully used using a new three-way calibration model for quantitative drug analysis in urine samples. A list of the most common nanomaterials/nanobiosensors used for the determination of opioids/opioids is reported in Table 4.

**Table 4:** List of drug abuse analyses carried out by electrochemical techniques, with the indication of nanomaterials/nanobiosensors, matrices, and limit of detections (LODs).

Reference	Drug abuse analyzed	Technique used	Nanomaterial/nanobiosensor	Sample	LOD (nmol/L)
[135]	Heroin, morphine, and noscapine	Differential pulse voltammetry	GNSs	Drug sample	400, 500, and 200
[35]	Codeine	Square wave voltammetry	GNSs/Nafion	Urine and cough syrup	15
[136]	Codeine	Square wave voltammetry	GNSs—CoFe_2_O_4_ nanoparticles	Blood, urine, and pharmaceutical tablets	11
[105]	Morphine	Linear sweep voltammetry	AuNPs	Urine and pharmaceutical tablets	0.43
[137]	Heroin	Capillary electrophoresis mass spectrometry	AuNPs	Drug sample	
[103]	Morphine	Differential pulse voltammetry	AuNPs	Urine	1.33
[138]	Morphine	Cyclic voltammetry	AuNPs	Urine	210
[139]	Morphine	Cyclic voltammetry and differential pulse voltammetry	AuNPs	Urine	4.21
[140]	Morphine	Cyclic voltammetry and differential pulse voltammetry	AuNPs/ferrocene	Urine	3.51
[141]	Codeine	Differential pulse voltammetry, cyclic voltammetry, and chronoamperometry	MWCNTs	Serum, urine, and pharmaceutical preparations	200
[142]	Morphine	Cyclic voltammetry	MWCNTs/Nafion	Serum	32
[143]	Morphine	Cyclic voltammetry and chronoamperometry	*N*-hexyl-3-methylimidazolium hexafluoro phosphate/MWCNTs	Urine and pharmaceutical injection	20
[144]	Codeine and morphine	Differential pulse voltammetry, cyclic voltammetry, and chronoamperometry	MWCNTs/SnO_2_-Zn_2_SnO_4_ Nanoparticles	Urine	9 for both
[34]	Codeine	Differential pulse voltammetry	SWCNTs	Urine, pharmaceutical tablets	110
[145]	Codeine and morphine	Differential pulse voltammetry	Zn_2_SO_4_ nanoparticles/graphene nanocomposite	Urine and pharmaceutical syrup	9 and 11
[146]	Morphine	Cyclic voltammetry	ZnO/CNTs	Urine	60
[46]	Morphine	Cyclic voltammetry and square wave voltammetry	Nickel oxide/carbon nanotubes (NiO/CNTs)	Urine and pharmaceutical sample	10
[36]	Codeine	Differential pulse voltammetry	WO_3_-CNTs	Urine	20
[37]	Codeine	Differential pulse voltammetry	Porous silicon/palladium nanoparticles	Serum, urine, and pharmaceutical preparations	300
[51]	Morphine	Cyclic voltammetry, linear sweep voltammetry, and square wave voltammetry	Pt nanoparticles	Pharmaceutical tablets	50
[38]	Morphine and codeine	Adsorptive stripping voltammetry	Pt nanoparticle/porous silicon	Blood and urine	30 and 20
[147]	Fentanyl	Cyclic voltammetry	Ru(bpy)_3_Cl_2_	Drug sample	8.5
[106]	Morphine	Cyclic voltammetry and linear sweep voltammetry	ER-MWCNTs-doped-GO	Blood and urine	200
[43]	Morphine	Differential pulse voltammetry, cyclic voltammetry, and chronoamperometry	MWCNTs/chitosan	Blood and urine	240
[103]	Morphine	Cyclic voltammetry and chronoamperometry	Vinylferrocene/MWCNTs	Urine and pharmaceutical sample	90
[51]	Codeine	Colourimetry	Citrate stabilized AuNPs	Postmortem blood	900
[104]	Morphine	Differential pulse voltammetry	AuNPs	Urine	1.33
[145]	Morphine	Differential pulse voltammetry	Zn_2_SnO_4_-GO/CPE	Plasma	11
[148]	Codeine, morphine, thebaine, oxycodone, noroxycodone, tramadol, and methadone	Colourimetry	AuNPs	Drugs in aqueous media and urine	–
[79]	Morphine	Voltammetry	BaFe_12_O_19_ NPs	–	20
[149]	Morphine	Differential pulse voltammetry	La^3+^-CuO/MWCNTs/CPE		8.0
[150]	Methadone	Differential pulse voltammetry	(PDDA)/MWCNT/CQDs	Urine and plasma	30
[151]	Morphine	Differential pulse voltammetry	RGO-Pd	Urine	13
[152]	Morphine	Cyclic voltammetry	OMC/GCE	Urine	10
[153]	Morphine	Cyclic voltammetry	AuNPs/GCE	Serum	41
[154]	Codeine	Differential pulse voltammetry	MWCNTs/ZnCrFeO_4_/CPE	Tablets, cough syrup, urine, and serum	9
[155]	Methadone	Cyclic voltammetry	Yb_2_O_3_NP-Ru(bpy)_3_^+2^-CPE	Urine	0.2
[156]	Morphine and buprenorphine	Differential pulse voltammetry	Rh NPs-MC/GCE	Pharmaceutical tablets	40
[157]	Methadone	Differential pulse voltammetry	(Gr/AgNPs)_2_/GCE	Blood	120
[158]	Morphine	Square wave voltammetry	FeWO_4_/CPE	Urine	580
[127]	Morphine and Codeine	Differential pulse voltammetry	dsDNA/MWCNTs–PDDA/PGE	Blood, serum, urine, and pharmaceutical formulations	600
[143]	Morphine	Cyclic voltammetry, Chronoamperometry	*N*-hexyl-3-methylimidazolium hexafluoro-phosphate/MWCNTs/PE	Urine and pharmaceutical injections	20
[125]	Morphine	Optical dipstick kit	AuNP-labelled morphine scFv-immunoprobe	Blood, urine, and saliva	20

GNS: graphene nanosheets; MWCNTs: multi-wall carbon nanotubes; CNT: carbon nanotubes; CPE: carbon paste electrode; RGO: reduced graphene oxide; OMC: ordered mesoporus carbon; GCE: glass carbon electrode; PDDA: poly dimethyl diallyl ammonium chloride.

## Conclusions

Classical and nanomaterial-based techniques are used for the analytical determination of opiates, each with their own advantages and limitations. Classical methods include colourimetric, spectroscopic, and chromatographic analysis, whereas nanomaterial-based techniques involve the use of nanoparticles-based biosensors and electrochemical sensors. Classical methods for detecting opiate have been widely used and are generally considered to be cost-effective, compared to nanomaterial-based methods which often involve the use of specialized nanomaterials and advanced technology, generally expensive to develop and implement. Electrochemical, nano, and supramolecular sensors are becoming essential tools for the detection of drugs due to their fast availability, reliability, and portability. Various techniques like conductometry, amperometry, voltammetry, and chronoamperometry are very versatile depending on the electrode type. These techniques provide high sensitivity and continuous monitoring of drugs, making them great tools for determining opioid intakes. The most used nanomaterials for electrodes are CNTs in MWCNT and glass wall CNT; however, platinum-based nanoparticles and AuNP show the lowest LODs. Moreover, the usage of nafion tubes has other advantages in the fabrication of biosensors for the opioid detection in biological fluids. Analysis time may vary between classical and nanomaterial-based techniques for the determination of opiates. Classical methods generally have relatively rapid analysis times, making them suitable for high-throughput screening; nevertheless, these methods require complex pre-sample treatment for drug extraction and concentration prior to analysis. On the other hand, although sample processing before analysis is not necessary for nanomaterial-based techniques, longer analysis times may be required compared to traditional methods, due to the complexity of the sensor fabrication, functionalization, and integration of nanomaterials into sensors, especially during the optimization phases, and the detection process.

In conclusion, both classical and nanomaterial-based techniques play important roles in the analytical determination of opiates. Classical techniques offer well-established methods, whereas nanomaterial-based techniques provide innovative tools for sensitive and continuous monitoring of opiates. New sensors can be considered promising technical devices, complementary to classical analytical systems, enabling fast, easy, and on-site drug detection, in specific preliminary analyses which need to be confirmed, at the moment, by classical methods. Despite the progress of an extensive spectrum of nanosensors over the last two decades, the future purpose of low-cost, high-throughput, multiplexed clinical diagnostic Lab-on-a-Chip instruments has yet to be fulfilled. Overall, the major advantages of nanoparticles in opioid detection are lower LOD, and portability; furthermore, nanosensors are particularly suitable for QuEChERS-based methods, representing a promising technology for the near future. Finally, it should be underlined that most of these new sensors contain materials and heavy metals that could be hazardous for human health and other living organisms. Therefore, safe handling along with data on the toxicity of nanomaterials is highly required.

## Authors’ contributions

Muhammad Usman contributed to conceptualization, methodology, and writing-original draft preparation. Yawar Baig contributed to methodology and writing-original draft preparation. Donatella Nardiello contributed to methodology, formal analysis and investigation, writing-review and editing. Maurizio Quinto contributed to conceptualization, methodology, writing-review, editing, resources, and supervision. All the authors contributed to the final text and approved it.

## Compliance with ethical standards

This article does not contain any studies with human participants or animals.

## Disclosure statement

None declared.
